# Oral health in an urban slum, Nigeria: residents’ perceptions, practices and care-seeking experiences

**DOI:** 10.1186/s12903-023-03303-5

**Published:** 2023-09-09

**Authors:** Mary E. Osuh, Gbemisola A. Oke, Richard J. Lilford, Jackson I. Osuh, Folake B. Lawal, Shakeerah O. Gbadebo, Eme Owoaje, Akinyinka Omigbodun, Babatunde Adedokun, Yen-Fu Chen, Bronwyn Harris

**Affiliations:** 1 Division of Health Sciences, Warwick, Medical School, University of Warwick, Coventry, UK; 2 Department of Periodontology and Community Dentistry, Faculty of Dentistry, College of Medicine, University of Ibadan, Ibadan, Oyo State Nigeria; 3 University College Hospital (UCH), Ibadan, Oyo State Nigeria; 4 College of Medical and Dental Sciences, Institute of Applied Health Research, The University of Birmingham, Birmingham, UK; 5 Department of Psychology, Faculty of Social Sciences, Federal University, Oye-Ekiti, Nigeria; 6 Department of Restorative Dentistry, Faculty of Dentistry, College of Medicine, University of Ibadan, Ibadan, Oyo State Nigeria; 7 Department of Community Medicine, Faculty of Public Health, College of Medicine, University of Ibadan, Ibadan, Oyo State Nigeria; 8 Department of Obstetrics and Gynaecology, Faculty of Clinical Sciences, College of Medicine, University of Ibadan, Ibadan, Oyo State Nigeria; 9 Centre for Observational Research, Amgen Inc, Thousand Oaks, CA USA

**Keywords:** Care-seeking experience, Oral health, Perception, Practices, Residents, Urban slum

## Abstract

**Background/introduction:**

One of the key recommendations for the new WHO global strategy for oral health is inclusion of disadvantaged populations and their engagement in policy dialogues such that their needs and views are addressed in policy decisions.

**Objectives:**

This study explored oral health perceptions, practices and care-seeking experiences of slum residents in Ibadan, Nigeria.

**Method:**

Focus group discussions (FGD) were conducted with family health-decision makers in an urban slum site. Oral health perceptions, practices, and care-seeking experiences were discussed. FGDs were recorded, transcribed, and translated. ATLAS.ti qualitative research software was deployed for analysis using thematic analysis.

**Results:**

Six FGD sessions, divided by gender and age, were conducted between September–October 2019, (*N* = total 58 participants, aged 25 to 59 years). Common dental ailments mentioned were dental pain, tooth sensitivity, bleeding gums, tooth decay, mouth odor, gum disease, and tooth fracture. Perceived causes of dental conditions included poor dental hygiene and habits, sugary diets, ignorance, and supernatural forces. Mouth cleaning was mostly done once daily using toothbrush and paste. Other cleaning tools were ground glass, wood ash, charcoal, *“*epa Ijebu” (a dentrifice)*,* and “orin ata” (a type of chewing stick). Remedies for relieving dental pain included over-the-counter medicines, warm salted water, gin, tobacco (snuff/powdered), cow urine/dung, battery fluid, and various mixtures/ concoctions. Visits to the dentists were mentioned by a few but this was usually as last resort. Main barriers to accessing care from dental care facilities were unaffordability of service charges and fear of extreme treatment measures (extraction). Suggested measures to improve timely access to dental health care included reducing/subsidizing costs of treatments and medications, offering non-extraction treatment options, and oral health education programmes.

**Conclusion:**

The slum residents experience various forms of dental ailments mostly pain-related. The residents perceived formal dental clinics as unaffordable, thereby engaging in self‐care remedies and harmful oral health practices before seeking professional help. Policymakers and decision-makers may leverage this empirical evidence for the people’s education on early dental care and address challenges to affordable, available, and acceptable oral healthcare services among slum residents to improve access to care facilities.

**Supplementary Information:**

The online version contains supplementary material available at 10.1186/s12903-023-03303-5.

## Introduction

One of the key recommendations for the new World Health Organisation (WHO) global strategy for oral health is the inclusion of disadvantaged populations and their engagement in policy dialogues such that their needs and views are addressed in policy decisions in efforts to reform oral health [1]. This significant resolution to strengthen the advancement of global oral health was made at WHO’s 2021 World Health Assembly. A typical example of a disadvantaged community is the slum, which is described as a densely populated residential area comprising mostly crowded, decrepit housing units in a situation of depreciated or incomplete infrastructure and occupied mostly by impoverished individuals [2, 3]. As the fastest-growing social cluster of communities in the world, slums provide homes to about one billion of the world’s population [2, 3]. In Nigeria, the slum population is reported to comprise about half of the entire country [4]. A major concern about slums is the effect of the environment on the overall health of the residents which manifests both as chronic and acute diseases [2, 5–10].

Oral diseases, like most other diseases, are profoundly influenced by people’s lifestyles and life circumstances [5, 11–13]. The prevalence of oral disease is worsened by the lack of attention paid to dental diseases by stakeholders in health when planning healthcare and service delivery especially within Low- and Middle-Income Countries (LMIC). This is a neglected epidemic according to WHO [14–16]. The global burden of oral disease is worsened by the grossly inadequate oral health work force in most countries [17]. In the hopes of improving access to oral health care in developing contexts in many countries, oral health has been integrated into Primary Health Care (PHC) systems [18]. However, in Nigeria, Community Health Workers (CHW), a part of whose role as ancillary staff is to provide preventive oral health services in PHCs are yet to be adequately positioned to bridge this gap [18]. Consequently, common oral diseases such as dental caries and periodontal diseases (PD) continue to increase in prevalence, especially among deprived population groups [19–22].

Dental caries and periodontal disease are both chronic infectious diseases of the oral region: while caries affects the tooth structure, periodontal disease (gum disease), affects the tooth-supporting structures. People living with oral diseases may experience a negative impact of these diseases on their quality of life, school performances, work productivity and family health spending [23–25]. Oral diseases are largely preventable, but up to 90% of populations surveyed from LMICs live with oral diseases that sometimes end in fatalities [26–28]. Our recent report of a higher oral disease prevalence among slum residents and their relatively poorer access to publicly funded oral health services [29] supports the burden of oral disease among disadvantaged groups. In the same study, we reported how cost of accessing dental services was a major determinant of use of available public dental services, since dental services are largely paid out-of-pocket. As such the dental health care needs of the slum residents are largely serviced by the traditional oral health care providers [26, 29–31].

The number of people residing in the slums of Nigeria and LMICs is growing [2], and the vast majority are living with oral diseases [29]. In order to provide acceptable interventions among such populations, efforts at promoting oral health and general well-being should be based on their perceptions about oral disease causation and prevention [1]. Similarly their oral health practices as well as their experiences in seeking care from existing care facilities, should be taken into consideration in planning intervention strategies [1]. Moreover, current evidence suggests an intimate link between slum settlement and the wider urban setting as the slums serve as transit points for many urban dwellers before they achieve better living conditions [3, 32–34]. Thus, any successful intervention for the control of oral diseases in the slum setting, is likely to have a far-reaching effect on the general urban populace.

Currently, few studies are available on the oral health of slum dwellers in LMICs and the existing ones are predominantly focussed on clinical outcomes [1, 29]. Qualitative studies on the beliefs and practices of significant gatekeepers have been conducted only in formal urban settings [30], however, little is known about the experiences of those living in slums, despite making up a large percentage of the population [4]. This implies that existing policy decisions in LMICs regarding planning and designing of oral health services may be without data on the perspectives and experiences of slum residents living with oral diseases [1]. The suitability and acceptability of the resultant approaches, so far, becomes questionable, more so for those living in slums. This study was therefore, conducted to explore the slum residents’ perceptions about oral diseases, their practices and care-seeking experiences.

## Methods

### Research design

This was an exploratory qualitative study using face-to-face focus group discussions (FGD) with representatives of households in Idikan, a slum community in Ibadan, Oyo State, South Western Nigeria. The study period was between September and October 2019. The study is a part of a broader research conducted to assess the prevalence and determinants of oral diseases and oral health care needs within different urban settings [22]. The aim of this study is to improve equity in access to oral health care services and improved oral health for residents of the slum. We were guided by the Access framework developed by Levesque et al*.* (2013) [35], which draws attention to multiple elements along an access pathway and their interactions across both the demand- and supply-sides of the health system. For example, this might involve a slum resident perceiving and acting on oral health need, seeking, reaching and paying for a healthcare service (e.g., as offered by a community dentist, a medicine vendor or a traditional healer), and adhering to the advice and treatment / preventative regimen prescribed (whether medically appropriate or not). Within this framework, access is mediated and enabled or impeded by wider social determinants such as health literacy, beliefs, culture, gender, and income, as well as the availability, affordability and appropriateness of healthcare sought [35]. Thus, our interest in ensuring an equitable oral healthcare service is closely tied to improving access to quality oral healthcare for slum residents, and with this, easing the access pathways within the local context. The research findings were reported in line with the Consolidated Criteria for Reporting Qualitative Research (COREQ) guideline [36].

### Ethics and consent to participate

All methods were carried out in accordance with relevant guidelines and regulations. As reported in our previous publications [22, 29], ethical approvals for the research protocol were granted both by the Oyo State Research Ethics Review Committee (AD 13/479/1247) of Ibadan, Nigeria and the Biomedical and Scientific Research Ethics Committee (BSREC: 37/18–19) of the University of Warwick. Permissions were granted by relevant stakeholders in the study site. Informed Consent forms were administered to participants and these were filled out before the start of research exercises in each Focus group session.

### Study setting

Idikan is a densely populated area comprising mainly people of the Yoruba tribe or ethnicity. It is located in the heart of the ancient city of Ibadan, 128 km northeast of Lagos. It has a landmass of approximately 14000km^2^ and a population of about 5500 [37]. The buildings are located along an old tarred road, wending towards the commerce beehive, consisting of contiguous medium scale markets, where many residents work as traders. The structures are permanent but mostly run-down, with poor sanitation and refuse-filled drains. The area is poorly planned with limited road network [37, 38]. In one of the buildings in the community is a small clinic—Primary Oral Health Care (POHC) centre that provides basic oral care and referral services to the community dwellers. Within the same building, the Primary Health Care (PHC) clinic is located. The two clinics are affiliated with a tertiary referral centre- the University College Hospital (UCH) which is about 4.5 km away making this relatively accessible to the slum residents in case of referral. Community Health workers (CHWs) are periodically posted from UCH to the clinics as frontline public health workers that promote health among community groups with limited access to health [39]. The CHWs in Nigeria include primary health care tutors (PHCTs), community health officers (CHOs), community health extension workers (CHEWs) and junior community health extension workers (JCHEWs) [39].

There are numerous patent medicine stores where individuals without formal pharmacy training sell pharmaceutical products to people with or without doctors’ prescription on a retail basis and purely for profit. Patent and Proprietary Medicine Vendors (PPMVs) as well as traditional healing homes abound in the area, all of which are accessible to members of the community [37, 38, 40].The structure of the community is such that it exists in compounds known in the native Yoruba language as "Agbo-ile" and is headed by a Baale (father of the compund). Agbo-ile is a collection of rooms or apartments occupied by different family or household members (ebi) [38, 41].

### Research team

The FGDs were facilitated by a team comprising a female dentist (consultant community dentist and the leading author) and an experienced male facilitator with over 10 years of practice on qualitative studies from a background in public health and social sciences. Both facilitators were conversant with the Yoruba Language. There was also an assistant (note-taker) who was a fresh graduate of Dentistry whose role was to observe non‐verbal interactions and the impact of the group dynamics, as well as to document the general content of the discussion, thereby supplementing the data from audio recording [42]. The team was trained on the details of the research and was fully involved in all research processes. The research team visited the community leaders to formally introduce themselves and establish a conducive rapport and to obtain their permission. Following briefing on the research conduct, copies of the "participant information leaflet”, a leaflet containing detailed information about the study and the researchers as well as the researchers’ contacts were given to them. All their questions were entertained and clarifications made. The team was in turn assigned a community gatekeeper, also a leader in the community, who facilitated the entire research process. We received a sampling frame which comprised a list of 40 existing compounds in the community which was reviewed by all stakeholders on site and adopted as current.

### Selection process and recruitment of participants

Given the homogenous nature of the community and the resources available to conduct the study, the authorsconsidered that a quarter of the total number of compounds that made up the community would be a fair spread and representation of the entire community. Therefore, 10 compounds (25%) in the community were randomly selected by balloting. From each of the study compounds, the “Baale” was designated the gateway to reaching desired participants. Using their knowledge of the compound, the Baale purposively selected compound representatives to participate in the study, guided by our inclusion criteria, namely: adults aged between 25 and 64 years, resident in the community for at least two years and decision makers on the health and wellbeing of the household, especially in oral health and care seeking activities. Participants had to be available for the research, willing to participate, and have the ability to communicate their experiences and opinions in an articulate, expressive, and reflective manner [43]. Letters of invitation were prepared in both English and Yoruba, and delivered in person. Explanations were also offered to those who could not read. The letters were accompanied by a copy of our “participant information leaflet”.

### Focus group discussions

The FGDs were conducted in one of the halls (a popular choice) within the community. Being in a central location within the community, the venue was easily accessible to all, and it was comfortable enough, private, quiet and free from distractions [44]. The FGD sessions were stratified along age group and gender: age group because of the culture of respect for elders that is widely practiced in the community, and gender because of the patriarchal system of the community [45–47]. All invitees turned up for their appointments. The size, age and gender distribution of the nominees, determined the number of focus groups sessions per stratum. Participants’ consent for voice recording was obtained [42] and the Informed Consent forms were filled before each session. Each session started with a brief introduction by the team lead. All facilitators were introduced by name and professional background. A brief form on sociodemographic characteristics (name of household and compound, age, sex, marital status, and whether or not they took decisions regarding the general health and well-being of members of the family or household), was used to generate data on participants socio-demographic variables. Support was provided for those that requested assistance in filling the forms. Thereafter, the participants were each given a pseudonym (a unique identifier) for anonymity during data collection.

### Process and data collection

A semi-structured discussion guide was used for all the sessions. The FGD guide was developed around concepts that were drawn from the literature, thus providing the basis for the key themes that cover the following spectrum: commonly experienced oral ailments, oral health practices, and experiences in seeking dental care. The guide was prepared both in the English and Yoruba Languages, pre-tested and validated before its use for the study. Open questions were supported with possible prompts that could be explored further. 

### Language

All participants were literate. The meetings were conducted mainly in the native Yoruba language as was the consensus. FGD sessions commenced with the opening questions, then the introductory questions and the FGD guiding questions [42, 48]. Facilitators encouraged participants to offer their own perspective, using prompts such as “what do you think?” “What are your views?” “Can we share your opinion?” etc. Responses were encouraged in each FGD session until they became repetitive, indicating saturation [42, 48]. Opportunity to validate the study findings (member checking) was made known to the participants, as a measure to enhance trustworthiness. At the end of each session (maximum of an hour) refreshments (a bottle of soft drink and a snack) were served. A tube of toothpaste and a toothbrush were also given to each participant in appreciation of their participation.

### Data handling

All audio recordings were recorded in digital format. These were encrypted and stored securely, backed up with password protection and then removed from the recorder. Recordings were transcribed verbatim, then translated into English by an expert. The English version document was given to another expert who back-translated it into the native Yoruba language and compared it with the original audio recordings for cleaning and correction. The transcripts were returned to participants who had indicated interest in validation of the study findings, for their comments and or correction. No feedback was received from any of them. The final document in English was then analysed using qualitative data analysis software ATLAS.ti (Qualitative Scientific Software, Berlin; V7).

### Data analysis

**Fig. 1:**
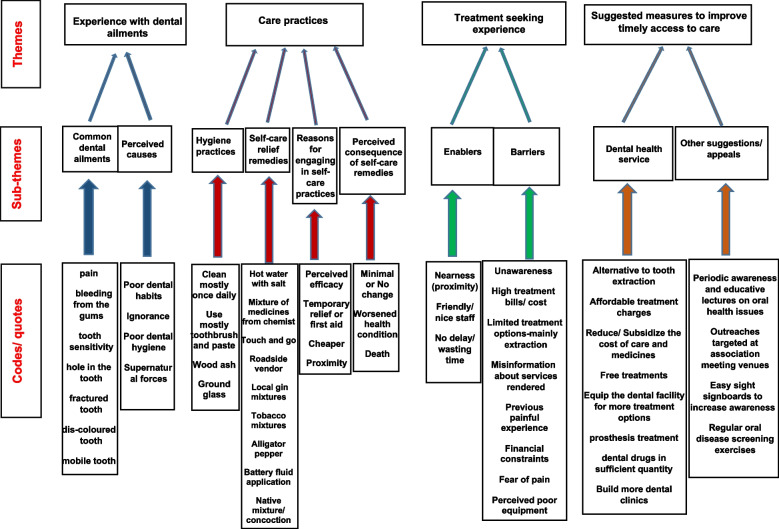
A diagram showing a summary of the themes, sub-themes, and codes from FGD with the slum residents on a map

Data analysis began with listening to the FGD audio recordings, followed by reading and rereading the verbatim transcriptions and translations [49, 50] until all “meaning units” were identified [51]. Then coding and re-coding of data items was performed to discover emerging patterns and new ideas which were repeatedly refined and some sub-themes became obvious. The themes and sub-themes were then described in a map to facilitate interpretation (Fig. 1). The key issues were teased out, the themes summarised and the data compared from across groups. The thematic analysis was structured around the key concepts in the FGD guide, while also allowing for emergent issues conducive to the development of new themes. The analysis process was carried out by two researchers independently and the results were compared and integrated.

## Results

A total of 58 representatives of household units were engaged in six focus group sessions, including five participants (each in a different FGDs) who were not originally invited but had accompanied invited members of their compound to the discussion (Table 1). Their inclusion did not seem to affect the dynamics of the groups. The participants aged between 25 and 59 years, were distributed evenly between each gender group. Forty of the participants were married, while two of them were living-in partners. Four were widows, eight had never been married and four were divorced (Table 1).

**Table 1:** Characteristics of participants (*N* = 58)

Characteristic	Sub-type	Number	%
Compounds		10	
Households	Families	53	91.4
	Accompanying compound members	5	8.6
Gender	Male	29	50.0
	Female	29	50.0
Age range		25 – 59 years	
Marital status	Married	40	69.0
	Living-in partners but not married	2	3.4
	Widowed	4	6.9
	Divorced	4	6.9
	Never married	8	13.8
Population per group	FGD1- Younger female	9	
	FGD2- Older female	11	
	FGD3- Younger male	8	
	FGD4- Older male	11	
	FGD5- Younger female	9	
	FGD6-Younger male	10	

All participants displayed enthusiasm. More vivid description of personal and family experiences about oral health were observed in the female sessions. The older men and women’s views were inclined more towards traditional oral health practices compared to the younger ones. Sessions lasted for an average of 50 min (range 40–60 min).

### Common dental ailments: perceiving a healthcare need

Pain of dental origin was by far the most common oral health problem identified by the participants. Most of the descriptions were about the intensity and severity of the pain, where pictures of extreme pain with severe or grave consequences – even death—were painted. A detailed list of the common dental ailments mentioned is available in Fig. 1.

*“This akokoro* (referring to toothache) *issues, hmm!* (shakes his head) *many of us have been disturbed by teeth pain o!”. FGD4_Older_MaleP4*

*“I have seen a lot of people that ‘passed on’*(died) *just because of teeth problems. Akokoro, is that not what they call toothache? Ah! if akokoro issues affect a person, or if you have seen someone that has akokoro, he or she will feel that he will not see or witness another day on earth just because of the pain”. FGD4_older_Male_P3*

The term “Akokoro” kept coming up in the discourse. Participants failed to reach a consensus on the condition “Akokoro”. While some referred to it as tooth decay or hole in the tooth, with or without associated pain, others argued that "akokoro” was the native name for gum diseases. Although argument in favor of tooth decay dominated most sessions, discussants generally agreed that “akokoro" is a dental disease associated with extreme discomfort. A few dissenting voices insisted that the specific cause of tooth decay was tooth worms, and was thus different from akokoro.

Bleeding gums also featured in the mention of common dental ailments.

*“At times when someone is brushing his teeth blood will gush out…. also whenever I want to brush my teeth blood will gush out” FGD4_older_male_P9*

Tooth sensitivity, albeit less common than pain and bleeding, was also identified by many of the participants. In describing the tooth sensitivity, some likened the feeling to an ache, a few others described it as an electric shock sensation elicited by acidic foods or drinks and water.

*“Shocking teeth, is a major issue, when I drink water, I feel it on my teeth”. FGD1_Younger Female_P8*

Dental caries (hole in the tooth) was also identified as a common dental ailment. While some referred to it as a hole in the tooth, others simply called it decayed tooth.

*“My own is not akokoro o! I had a piece of meat stock in between my teeth then I tried to remove it using an object to force it out, it now left a hole under my tooth. That gave me the sign that I had damaged my tooth as a sharp pain followed subsequently, it was a small hole initially, then, suddenly it became a wide and deep hole. So, I went to the hospital and they took a picture of it and showed me how deep the hole was. That was caused by the teeth worm, so it is not akokoro* (tooth decay) *that caused it* (the participants affirms)*”. FGD2_Older_FemaleP5*

Fractured teeth and mobile teeth including discoloured teeth were also mentioned.

*“The problem I had with my teeth in the past was that my tooth got broken from the gum”. FGD6_Younger_MaleP3*

*“My own issue was I use to have a slack* (mobile)* tooth. It used to pain me whenever I ate with it. I have waited for so long for the tooth to heal but to no avail, so I went to the dentist to remove it for me” FGD4_Older_MaleP11*

Further details of common dental ailments are contained in Appendix 1.

### Perceived causes of dental ailments: health literacy and beliefs

Most people mentioned poor dental hygiene as a major cause of dental health problems.

*“I think for those children (referring to those with poor dental health) of ours, it is because they don’t wash their teeth well, that is why it bleeds. if they wash their teeth well, it will not bleed” FGD2_Older_FemaleP11*

Many discussants traced the foundation of poor dental hygiene and formation of poor dental hygiene habits to childhood, they blamed the parents or guardians for setting people on a path of bad hygiene that persists throughout their lives.

*“Poor parental guidance too contributes greatly to the dental problems commonly seen today” FGD1_Younger_FemaleP3*

In addition, others opined that the causes include: ignorance about appropriate dental health practices, excessive consumption of sugars.

*“My daughter has dental problems as we speak and I know it is caused by her excessive sweets and sweet foods intake, even chewing gums. Her mouth is always busy. She indulges so much in anything sweet”. FGD1_Younger_ FemaleP1*

Some participants blamed deities or supernatural forces for inflicting dental disease.

*“Hm! When the powers that be takes offense in one, they can hit their victim using something as simple as smelly mouth, simple but that has the capability of preventing the person from progressing in life. May we not offend those that cannot forgive us in life” FGD3_Younger_ MaleP2*

Other people believed that dental diseases are inherited in the family.

*“As for me, I believe dental problems are inherited. If you look well, you will see a pattern in the family” FGD6_Younger_MaleP9*

Further details about participants perception of the causes of dental ailments are contained in Appendix 1.

### Dental health care practices: personal and social values, knowledge and available care within the living environment

Dental hygiene: It was clear that most of the household representatives understood the importance of mouth cleaning as a good oral hygiene practice.

*“when we wake up, it is a must to brush our teeth, some people use brush with close up* (a type of toothpaste). *I personally, use brush and close up. You know, there is no one who will wake up and not want to care for their mouth in the morning. Before you eat, even if it is pap* (soft or semi-liquid food usually made from grains of corn)*, you must brush your teeth first. That is the way I understand it”.* FGD2_Older_FemaleP2.

Cleaning frequency: Mouth cleaning was generally done once daily as the household representatives in each session unanimously agreed, in principle, to the once-daily mouth cleaning.

*“When we clean our mouth, first thing in the morning and do it well with whatsoever mouth cleaning tool is used in the family or compound, we are good to go”* FGD3_Younger_MaleP7

Facilitator: do you also repeat mouth cleaning in the night?

*“No!!, why? there will be no need for that. I know the teaching is different, but it all has no meaning. Just wastes!...*

Teeth cleaning implements: Different mouth cleaning implements and local dentrifices were mentioned and these included mixtures of ground glass with wood ash.

*“A mixture of ground glass with wood ash is effective for cleaning the mouth, even when you grind ordinary charcoal, it is as good”* FGD1_Younger_FemaleP3

*“We also use “Orin ata”. It is a chewing stick. It is spicy and if you are using it to brush, you will feel its hotness. It cleans the mouth thoroughly and prevents diseases* FGD2_Older_FemaleP1

Further details about participants dental hygiene practices are contained in Appendix 2.

### Relief remedies for dental ailments

Various categories of relief remedies for oral health problems were mentioned by the residents. A detailed list of this can be found in (Appendix 2). The relief remedies ranged from self-medication using various non- pharmaceutical mixtures, to visiting a dental clinic.

*“When my tooth pained me, I didn’t go to the hospital then o! I went to a chemist for treatment instead, and I was given a mixture of medicines. Plenty like this, I don’t know the names…” FGD3_Younger_Male_P8*

*Sometime when my tooth was disturbing me … and I was feeling the pain continuously. I applied the teeth medicine, the one they call "Touch and go". Since then, I never felt any pain again, to date. FGD5_Younger_Female_P9*

There were also a range of alcohol mixtures, tobacco mixtures and a range of other food products mixed together with some substances, all deployed as traditional remedy for the relief or cure of dental related problems.

*“We sometimes use ‘didi, asa’* (tobacco- snuff) *Yes!* (she affirms), *it is placed on the teeth where it hurts, and that’s it” FGD2_Older_Female_P7*

*“When my tooth issue started with me. The pain was serious. Someone advised me to get local gin and hold it at the corner of the tooth for some time and it worked”. FGD1_Younger_FemaleP2*

*“I know of a mixture of salt and alligator pepper; it helps” FGD4_Older_Male_P10*

Some dental pain remedies were said to be prescriptions prepared by the traditional healers.

*“When I had dental pain, I used traditional soap *(native black soap mixed with other things and usually prepared or prescribed by a traditional healer)* to cure it. I didn’t go to any hospital.*

Some alleged that battery fluid was helpful in the treatment of dental pain.

*“I hear some people use battery fluid for tooth ache problems. I haven’t had reason to try it though, but I know it can also be used for whitlow* (a painful and highly contagious boil or infection on the fingers) *to dry it. But you must be careful to not use too much of it* (referring to the battery fluid) *in any of these cases” FGD1_Younger_Female_P2*

The use of dental clinic services was often as a last resort, after other remedial options failed.

*“I now go to* [name of location] *whenever there is need for dental care, after our native methods failed. Even the one or two times my grandchildren had need for dental care; I counselled my daughter to take them to [name of location]. As a matter of fact, I follow them there* (referring to dental clinic)*” FGD3_Older_MaleP7*

### Basis for the choice of relief remedies

People sought remedies that were affordable, available and effective. Immediate or instant relief was more attractive even though some knew the effect would be short-lived.

*“When I was feeling a terrible pain in my tooth, and I couldn’t bear the pain anymore and the chemist was nearby. FGD3_Younger_Male_P8*

For some discussants, confidence in the efficacy of the remedies was the main reason, while for others, that the self-remedies were a cheaper option than seeking care at a dental clinic facility was paramount.

*… yes of course there are changes, improvement even, I felt better, if not, I will not be able to stand here today. The mixture is very effective and affordable FGD4_older_Male_P9*

*“When I used the native medicine* (from the traditional healer)*, it worked very well for me. That is why I shared it with others who had dental problem to use and they also testified to its effectiveness. Even the traditional soap, I gave some of it out to help someone who had dental related problem and the person got a relief. So, these things work, don’t under-estimate them o!”. FGD6_Younger_Male_P3*

Further details about participants basis for choice of relief remedies for dental pain are contained in Appendix 2.

### Perceived consequences of self-care remedies

The FGD also explored participants perceived effects of the self-medication. Some participants identified positive consequences (included in earlier section), but others noted negative consequences including: doubts about efficacy as well as disappointment.

*“Yes, I used the native treatment but there was no change because the pain didn’t go. It just provided relief for about 15 to 30 minutes”. FGD4_Older_MaleP3*

*“Me, I have used the traditional treatment and known how it works. It is better for someone to make a wise choice in seeking appropriate treatment from a dental clinic because I have personally received several disappointments from native or traditional treatment methods on my dental problems” FGD3_Younger_MaleP9*

A participant acknowledged that he did not experience any relief from pain until he took paracetamol.

*“Thank you. I had a similar experience with the previous person that spoke, it happened *(referring to dental pain)* to me too and they advised me to use a native mixture of local gin with Atare* (alligator pepper)*. It was just like drinking ordinary alcohol. It didn’t work at all. So, I decided not to use the native treatment again. But when I used ordinary paracetamol, the pain disappeared. Even when I developed a swelling afterwards, I still used paracetamol and it went down. So, I have stopped, I will not use the native treatment again”. FGD3_Younger_MaleP6*

Fears about the likelihood of some of these remedies causing harm to some organs in the body and even worsening the oral health condition were also expressed. Some discussants explained that death could result from indiscriminate use of certain remedies. Across all FGDs, there was general agreement that death could be an outcome of using battery fluid and was to be discouraged.

*“That was the same thing one of my sisters did and died, the husband complained about the tooth pain the wife was going through, then he was advised to use the battery fluid, she applied it and it corroded her intestines, that was how she died,…… since then no one uses it again”. FGD6_Younger_FemaleP7*

*Our people believe that battery water kills dental disease. But it is at a high risk. So, it is not advisable to use battery water. FGD4_Older_MaleP1*

Further details about participants perceived consequences of use of self-care remedies are contained in Appendix 2.

### Treatment-seeking experience: the enablers and barriers to reaching and utilising oral care facilities

#### Subtheme- the enablers to reaching and utilising oral care facilities-

Closeness of the facility to the people was identified as an important enabler to accessing dental care. The fact that a government dental care facility was present in the community was given as a notable illustration. A warm welcome and friendly disposition by clinic staff towards clients, as well as prompt response and timely service delivery from care providers were identified as enablers of utilization of dental care services or for subsequent visits.

*“What I like about this place* (dental centre) *is its closeness. But whenever we are referred from here to the…or …* (mentioned names of teaching hospitals and secondary health care facilities)*, hmmm! Those places are far o! The teaching hospital in particular is far, and very stressful, this place is better, please!” FGD3_Younger_MaleP2.*

*“During my own visit, not only were my teeth well treated, they also lectured me on the ways to manage my oral health immediately after treatment and after healing”. FGD3_Younger_MaleP7*

#### Subtheme- the barriers to reaching and utilising oral care facilities

Participants decried the limited scope of care being offered, they had thought the reason for referral was because the facility couldn't manage the cases and this therefore indicated poor quality. Lack of money was identified as a major barrier to accessing dental care in care facilities. The participants’ responses seemed to suggest that the total cost of dental treatment was usually unaffordable. Even some previous dental service users identified unaffordable costs as a barrier to re-accessing care. One discussant identified multiple payment points for items such as for card, consultation, treatment, and drugs as separate payments contributing to the huge bills incurred.

*“It is because of money o! You know, when you don’t have money, you can’t do anything. Someone has said it here earlier that he could not further his treatment at the hospital* (the dental service from the hospital)* all because of money. So, no money is the major factor” FGD3_Younger_MaleP6*

Discussants suggested that a painful previous experience discourages repeat visits and possibly encourages patients to resort to alternatives during subsequent dental care needs.

*“…money too is a serious challenge, but there are times when one wish to seek further care from the clinic but fear of a repeat of a painful experience from the last visit discourages one, my previous experience was not pleasant.” FGD2_Older_Female_P1*

Participants expressed dissatisfaction that tooth extraction often seemed to be the only remedy for any and all complaints presented at the existing dental clinic in the community, hence the clamor or request for treatment modalities other than extraction. A discussant specifically presented the plea on behalf of others in the group for more robust, non-extraction treatment options. Although they could not name the specific services, they maintained that any measure to retain their teeth in their mouth was better than extraction. Furthermore, it was suggested that an alternative to tooth extraction would eliminate the fear associated with pain, thus increasing appropriate dental care seeking behaviour within the community. This view was widely held by discussants in most FGD sessions.

*“we don’t want to remove our teeth again. …We want other solutions, something that can stop the pain but keep our teeth in our mouth and affordable too. They should please assist us in our community”. FGD4_Older_MaleP4*

Lack of awareness of the existence of a dental care facility in the community pervaded most FGD sessions. However, some participants pointed out that it may be new residents in the community who were unaware of the presence of a clinic.

*“I don’t agree, there is no one that can confidently say that he or she is not aware of dental clinic services in our community except the person who just moved into the neighbourhood. Well, it all boils down to the same thing. Please help us out with this periodic awareness programme” FGD2_Older_FemaleP9*

Limited awareness and some degree of misinformation about the function and types of services available at existing government clinics, constituted a major barrier to seeking dental care.

*To me, it didn’t appear they could offer much* (referring to varied services) *asides extraction. So, I just never bothered visiting the place. FGD 5_Younger_MaleP9*

Further details about participants care seeking experience under the enablers to accessing care from care facilities, are contained in Appendix 3.

### Suggested measures for improving timely access to dental health care: Acceptable, available, affordable services for slum residents

The need for a better equipped dental care facility, with dentists from different sub-specialties, for a wider range of services, spanning prosthetic tooth replacement to a more robust service delivery system within the community, was expressed.

*"We need more dental health care centers and doctors that are experts in different parts of the mouth* (dental sub-specialties) *to give us treatments that will prevent us from removing our teeth and the kind of treatment that will cure dental pain forever in our community”. FGD4_Older_MaleP4*

*"We want plastic teeth made in our clinics here too. If we can have it here, it will be so good, but you know, it is easier getting it from here than we go for it in…* (named teaching hospital)*” FGD4_Older_MaleP1*

Discussants explained that community members would make better use of the clinic if the cost of care was reduced while a few others wanted dental treatments to be provided to them at no cost at all.

*“…We are not saying don’t collect money, just that it shouldn’t be too much… and then they should not collect money before treatment, treat first before you ask for money” FGD2_Older_FemaleP2*

*“I think the cost of treatments should actually be made free” FGD2_Older_FemaleP11*

The desire to have readily available, affordable or discounted medications and prescription drugs within the clinics also featured in most discussions.

*“I have nothing except those that are in charge should ensure that there are enough clinics with sophisticated equipment and drugs at affordable prices, once we get to the hospital for treatment there should no delay, we want to receive an instant treatment to ease our stress”. FGD4_Older_MaleP3*

Visible signage to increase awareness about the existence of the government dental health facility was recommended in most sessions.

*“Me, I don’t think there is a signboard to inform or direct people to the dental clinic in the neighbourhood. If there is then it must be small and hidden. I suggest that a very big one be done, and placed in a strategic place to notify people of the availability of dental health services and activities here and possibly other small ones* (signboards*) to give direction. I believe this will improve awareness” FGD6_Younger_Male*

Participants were unanimously in favour of increased oral health literacy as the majority of them admitted to having limited knowledge on oral health matters. Many participants requested more engagement with the dental health care team through their various sub-population groups and associations as well as through home visits. This in their opinion, should improve community awareness of oral health matters.

*“Like ma, this type of discussion on the oral health issues of our people, we are having was because you invited us. En!* (Affirmative)*, that is what we want, from time to time”. FGD2_Older_MaleP4*

Periodic dental health screening to identify those in need of care and to avoid late diagnosis/presentation was also suggested. A discussant said he was not aware of the need for a biannual check-up at the clinic, adding that community members would be willing to embrace preventative services.

*“Oh, I see, we are supposed to come to the clinic for our checkup once in six months, ehen! I didn’t know o!. Any action that can help prevent dental problems is worth the trouble. FGD4_Older_MaleP6*

Further details about participants care seeking experience under the barriers to accessing care from care facilities, are contained in Appendix 3.

## Discussion

Achieving access to essential oral health-care services and oral disease prevention for slum residents requires impactful policy solutions and decisive upstream action on political, social, environmental, and commercial determinants of oral health [1]. Yet, there is a dearth of published evidence both in Nigeria and in the LMICs on the voices, perceptions, and preferences of the people who live in slums about their oral ill health. The global strategy by the WHO’s 2021 World Health Assembly resolution [1], recommends the inclusion of such information in policy decisions for sustainable interventions to improve the oral health and well-being of the slum residents. Guided by Levesque et al.’s. (2013) Access framework [35], our study sought to better understand the experiences of slum residents living with oral diseases along pathways to oral healthcare: common oral diseases, perceived causes, oral healthcare practices and oral ill-health relief remedies, the barriers and enablers they face in seeking dental health care from care facilities; and identify their preferences that can facilitate timely access to dental care.

Pain was identified as the most common dental ailment experienced by many, a finding similar to reports from clinical outcome studies conducted in LMICs and globally [29, 52]. This is, even as the residents made efforts to enhance their oral health and prevent diseases such as mouth cleaning, which was usually done once a day and in the mornings. Toothbrush and paste ranked most prominent among the tooth cleaning implements they deployed, although the use of traditional cleaning implements such as "Orin ata"—the roots of Fagara zanthoxyloides (a type of chewing-stick), "epa Ijebu" (a local dentrifice), ground charcoal or wood ash was preferred by many because of their perceived medicinal properties and less cost compared to the toothbrush and paste. Studies elsewhere have similarly reported a range of cleaning implements which varied from seemingly harmless to potentially harmful substances: charcoal and “miswak” in Tanzania [53], cotton wool, salt and water only in Nigeria [54], charcoal, sand, snuff powder, “neem”, twang in India and Tehran [55–57]. However, to the best of our knowledge, the practice of grinding glass (bottle) for use as a mouth cleaning implement has not been reported in other country contexts.

For the relief of pain or dental treatment, all participants irrespective of age group used a variety of self-care remedies. These include over-the-counter medicines, gin, tobacco and cow urine/dung, and concoctions including various preparations / mixtures which may be fatal eg. battery fluid or potentially hazardous e.g., “Touch and go”. “Touch and go” is a medicinal toothache oral solution prepared as red liquid commonly deployed for temporary relief of pain caused by mouth ulcers, denture irritation, and teething in children [58]. It is found readily available in retail outlets within and outside the slum environs. The liquid contains two active ingredients, clove oil 3.12%, which is a local anesthetic agent, and Tolu of balsam, 1.25%. Other constituents are menthol 1.25%, solvent ether 1.5%, phenol 1.25%, cajuput oil 2.5%, and vehicle to 100% [58]. While “Touch and go” provides temporary relief for tooth pain, its phenol content is capable of causing irreversible damage to the pulp leading to pulp necrosis [59]. Studies investigating the use of dental self-care remedies among slum populations in the LMICs are scarce. Available studies have examined the same from the general urban population. Findings from parts of Nigeria have identified the use of a saline wash, herbal preparations, antibiotics, and battery fluid as self-care remedies [30]. In Cameroon, substances such as petrol and vinegar, tobacco, urine, alum, ice-pack, and 'Touch and go' were used as self-care remedies [60]. In Sudan, commonly used self-care remedies included cloves, herbal remedies, and 'over the counter' medicines [61] whereas in Tehran, baking soda dissolved in water, warm salt water, and boiled sumac are commonly used as dental self-care remedies [57]. Consistent in all these studies is the fact that use of dental services from care facilities was practiced by few who considers it a last resort – when tooth extraction may well be the most appropriate treatment for the presenting condition [62, 63]. The reported dissatisfaction with ‘extraction only’ services in this study was clearly connected to the perceived associated pain from the extraction, hence the appeal by residents for other treatment modalities. Contrary to this perception, there are a range of specialty services available for dental care: periodontist (gum specialist), prosthodontist (specialist in dental prothesis), endodontist (root canal specialist), orthodontist (those who specialize in teeth alignment), paedodontist (pediatric dentist), the maxillofacial surgeons to mention but few. Although a few of these services such as orthodontics and major maxillofacial surgeries are available only at the higher levels of care (secondary and tertiary centres), appropriate referral system may be activated from the primary oral health care settings.

Late presentation at the dental clinics may also be partially explained by the silent nature of many oral diseases such that the sufferer may not be aware [26, 28, 64–66]. Consequently, the disease is neglected, until complications set in [67, 68]. Even in situations of mild, symptomatic dental problems, the fact that many people do not associate fatality with oral diseases often leads to a delay in their seeking of care [69].

The wide belief in the efficacy of self-remedy options, its ready availability as well as cheaper cost, relative to care received from care facilities are consistent with reports from other studies [60, 61, 70] and indicate a wide-reaching practice beyond the slum community, which has implications for oral health policy beyond slum settlements.

The expensive nature of dental treatment is a barrier to accessing oral health care globally. Owing to the lack of robust medical insurance and limited resources, particularly in the LMICs, many dental treatment procedures have been considered luxury [13]. In low-income communities such as slums, paying for dental services may be a low priority relative to the struggle for food and shelter [71]. It is well known that the traditional treatment for common dental diseases is costly relative to the treatment of other common systemic diseases even in high-income countries [13]. Unaffordability of care has been identified as barrier in many studies which looked at the role of cost in dental healthcare-seeking behaviour [29, 67, 72, 73]. It is therefore not surprising that the slum residents suggested reducing the cost of securing dental treatment, making dental treatments and medications free of charge or at best at subsidized rates as ways of enhancing their timely access to dental health care.

### Implication for policy and practice

Pain, being the commonest dental ailment expressed by the people, has implications for prevention. A context appropriate oral health education programme is needed to provide education and information as well as affordable services to slum residents to encourage timely access to care. The involvement of CHWs as well as Baales or the household heads should be considered in planning dental care programme at the community level as part of the community strategies to reach and or educate the residents. The CHWs in particular have been reported to be highly effective and contribute meaningfully to progresses in community health [39, 74, 75].

The residents needs to be encouraged to seek care early to avoid the complications that ultimately reduce their treatment options to extractions [23]—the reason why they perceived that tooth extraction was the only service provided in dental clinics.

Misinformation about perceived causes of oral diseases (supernatural forces) will need to be corrected. Similarly, the general resident population should be enlightened about the dangers in some oral care implements such as ground glass. All residents, patent medicine vendors, including dealers in the hazardous remedies commonly deployed for relief of dental pain such as local gin, tobacco, “Touch and go” battery fluid, cow urine/dung, various mixtures and concoctions, should be made aware of the potential dangers in the use of such as remedies for dental diseases.

The use of self-medication should be completely discouraged. This is because the condition being considered for self-care may be wrongly diagnosed, the drug dose may be inappropriate, medications may be contraindicated, toxic, harmful, or involve adverse reactions [76]. Building oral health educational content to include lessons from this research may better encourage a sense of responsibility and empower the people to take control of their own oral health.

The National Environmental Standards and Regulations Enforcement Agency and the State ministries of Environment are encouraged to tighten regulations around access to hazardous substances such as the battery fluid. Anecdotal evidence suggests poor enforcement of such regulations given by the unhindered access to battery acids (fluids) in Nigeria’s open markets. Appropriate standards and policies should be established around vulnerable populations and communities where the use of battery acids (fluids) can be abused such as extending it for use for tooth pain relief or other body infection (whitlow). Such measure may further reduce the incidence of acid bath vendetta that is sometimes seen in similar communities [77, 78].

Policymakers and those planning dental healthcare should consider strategies that can encourage or promote use of timely and preventive dental health care seeking specifically at the community level for slum environments. Access to dental healthcare particularly in terms of cost and affordability need be ensured to encourage increased use of dental healthcare services from care facilities. For example, the community based health care insurance may be repositioned to include dental services in order to address cost of accessing dental health care [79, 80]. Periodic training and re-training for all CHWs on oral health promotion and prevention may be considered as a part of their curriculum and job description in order to utilize them for preventive oral health care services [18]. It is hoped that these steps would encourage timely use of preventive dental health care services.

### Relevance of finding for WHO global strategy for oral health

In reporting the voices, needs, and preferences of residents of a disadvantaged community (slum), our study brought to focus, the broader determinants of health that shape the slum residents’ lives and health which are generally absent in the planning and designing of oral health intervention strategies [1, 81]. It is hoped that the information generated will facilitate the achievement of a sustainable and affordable access to essential oral health-care services and disease prevention among the people [65].

### Strengths and limitation

The approach adopted during selection process, of which a quarter of the compounds in the sampling frame was chosen by random technique, afforded diversity of context and ensured representation and adequate distribution of the residents.

The purposive selection of participants through nomination by each compound head (Baale), meant reliance on the Baales’ sense of judgment, alone, about who possess the desirable attributes for inclusion. This is a potential source of bias (in judgment), however, considering that no particular motive was noted during discussions with the Baales after the selections were made, this was not much of a concern. The purposive sampling method, afforded us the opportunity to gather a rich yield of information from the FGDs.

The enrolment of adults, who control their household resources was because of their influence in taking their own health decisions and that of their household members [82, 83]. We recognise their perceptions, challenges and suggestions as important information for policy decisions as equal partners in efforts to reform oral health care [1] as perceptions are well-known important drivers of health-related behaviours [37, 84]. Additionally, the participants’ residing in the slum confers on them knowledge about the environment.

We intentionally engaged diverse groups according to gender and age to overcome the patriarchal dominance and culture of respect practiced in the community which may stifle expression of subsets of the population [45–47]. Furthermore, facilitation of all FGD sessions were conducted by male and female as we thought this was important to make participants in either gender groups feel more comfortable [45]. This was strengthened by the repeated assurances to the participants that the research was about them and that we, as researchers, only wished to hear them out.

As a well-known Community Dentist in the study area, and one of the FGD facilitators, participants may have overtly wanted to take part in the research to please me (lead author). This is recognised as a potential source of bias as they may have sought to participate only in a positive way. Offsetting the potential bias introduced by my professional background and experience, necessitated engaging the services of the experienced group facilitator who was non-dentist and it helped. Being a male, his presence made those participating in the male groups in particular, more relaxed.

The FGD process was such that each participant had ample opportunity to contribute, allowed differences of opinions to be voiced fairly, prevented domination of discussions by one member (by laying emphasis from the outset, the importance of hearing a range of views), and encouraged reticent participants to speak [44].

As one of the facilitators, the lead author’s own pre-conceptions of the phenomena under study were identified, which were shaped by her professional experience as a dentist, general assumptions and cultural factors. These factors, which could influence the data interpretation, were suspended as much as possible by consciously inhibiting own meanings and interpretations (bracketing) in order to enter into the world of the participants [85].

To minimize subjectivity in the coding and minimize bias in the interpretation of the data, two authors (GO and BH) who were also experienced in qualitative studies evaluated the process and supervised the conduct of analysis. All three researchers independently checked the codes and themes and held discussions until consensus was achieved to establish credibility.

Overall, this study is the first to explore the perspectives of slum dwellers in the LMICs about their oral health needs, their perception, practices, and experiences of oral health, and care-seeking.

## Conclusion

The commonest experience of dental ailment in the slum is pain. Although there are cultural considerations around the beliefs about dental pain, their causes and how to remedy this, the main barriers to seeking care from care facilities are with affordability, service availability and delayed care seeking, which fuels anxieties and fears. Some of the self-care options adopted as remedy for dental ailments are potentially harmful and hinder timely care seeking from care facilities. Targeted oral health promotion and preventive education is required to encourage timely use of dental services from the care facilities, discourage the use of locally sourced tooth cleaning materials as well as discourage self-care remedy alternatives to formal dental care services. Policymakers and those planning dental healthcare should consider strategies that can promote access to dental healthcare, particularly in terms of cost and affordability and service availability for example: community-based healthcare insurance, and a better equipped dental care facility for a wider range of services.

### Supplementary Information

**Additional file 1.** 

**Additional file 2.** 

**Additional file 3.**

## Data Availability

All data generated or analysed during this study are included in this published article. No additional data are available.
