# Supplementary material for: Oral health in an urban slum, Nigeria: residents’ perceptions, practices and care-seeking experiences
Source: BMC Oral Health. 2023 Sep 9;23:657. doi: 10.1186/s12903-023-03303-5 (PMC10492367; doi:10.1186/s12903-023-03303-5)
Supplement: Supplementary file 1 — Additional file 1. [file 12903_2023_3303_MOESM1_ESM.docx]

**Appendix 1: Slum dwellers’ experiences of common dental ailments and the perceived causes**

| ***Theme*** | ***Sub-theme*** | ***Household representatives’ quotes*** |
| --- | --- | --- |
| ***Slum dwellers’ experience of common dental diseases*** | Pain | *“If akokoro grabs (affects) anyone, the person will agree that God does not have two names! (a phrase commonly used to describe someone in deep trouble). The mouth will be swollen with pus discharge. The pain is usually much” FGD3_Older_MaleP12g`* |
|  |  | *“What I know about that tooth decay is that, it is not something you can bear, my mother had it, there was a day she called us and told us that the pain had become unbearable and she is about to lose her life”. FGD2_Older_female_P10* |
|  |  | *"Regarding mouth issue, I don't have any, but I have an issue with my teeth, whenever I am chewing I always feel the pain under my tooth so even just of recent, I went to remove it because I was not feeling comfortable”.*  Facilitator: (laughs) so how many teeth have you removed now*?*  *Respondent: “Up to like four teeth”. FGD2_Older_FemaleP1* |
|  |  | *“I have seen one of our brothers that had akokoro issues it resulted in a big(swollen) mouth”. FGD3_Younger_MaleP2* |
|  | Gum bleed and other forms of mouth discharge | *“My first encounter with teeth issue, there was a time when I finished brushing my teeth and when I pressed the gums it will bring out blood and some particles without paining me” FGD5_Younger_female_P2* |
|  | Electric Shock-like sensation (tooth sensitivity) | *“What I noticed on my teeth was that whenever I take or eat oranges my teeth, it will start paining me seriously. Also if I drink cold soft drinks like coke and others, if I drink anything that has a lot of lemon inside my teeth will be paining me’. FGD4_older_Male_P1* |
|  |  | *“Yes I have experienced dental pain and people around me too, so it is not new to me at all, I had akokoro (Toothache) issues to the extent that when I drink water, I will feel pain and I am over 40 years now” FGD1_Younger_Female_P2* |
|  | Hole in the tooth | *“Akokoro is very common o! not just with us in here (referring to the community), I believe everywhere too! As it affects the adults, the children are not spared. I can say it is the main dental problem sa! That all the teeth will have spoilt (decayed)” FGD5_Younger_FemaleP10* |
|  | Fractured teeth | *“For me, I observed that two out of the remaining teeth in my mouth had broken. It got to a time that whenever I ate something with that side of the mouth, I would feel pain. The two teeth started paining me more, if something touched the two teeth, it would turn to a headache. So I went to the dental clinic in …(named) and they removed it for me and I am better till now…. FGD4_Older_MaleP7.* |
|  | *Discoloured or black teeth (dental stains)* | *"The teeth are very important and add beauty to the body. Some people are less than 40 years and their teeth are blackish, it is not good looking at all, it should be that, when you wake up in the morning, you should brush, but some people will say that 'when you wake up in the morning, brush' and do not specify if you should brush in the morning or night, but it is good to brush the teeth clean. What even, is the cause of the black teeth?” FGD1_Younger_FemaleP3.* |
|  | *Mobile teeth* | *“My own issue was I use to have a slack (mobile) tooth. It used to pain me whenever I ate with it. I have waited for so long for the tooth to heal but to no avail, so I went to the dentist to remove it for me” FGD4_Older_MaleP11* |
|  |  |  |
| ***Perceived causes of dental problems*** | Poor dental hygiene and habits | *“I use my teeth to open drinks. My friends and I am sure, many of us seated here do too. It was only recently that I felt my tooth crack while opening a bottle of drink, and that marked the beginning of my dental problem. It progressed from the feeling of shock to pain, and then involved many teeth”. FGD2_Older_FemaleP5* |
|  |  | *“…Some of us use our teeth to open soft drinks. This has serious implications. I have seen someone that used his teeth to open a soft drink and immediately he had jaw dislocation. Initially, he was self-medicating, the native way. Later, his whole mouth was affected and when they took him to the hospital the doctor said they will operate his mouth. Just to open a drink of #70, he had to pay #700,000 for surgery, to correct the dislocation…. FGD4_Older_MaleP5* |
|  | Low level of awareness about appropriate dental health practices | *“… when children start teething, I think it starts from there. When these children are about 6 months, and they start giving the child food, they should also use cotton wools to clean the mouth of the child from this point, they should not wait till the child has erupted all the teeth, by then it may be too late”*  *Moderator: Is that ignorance too?*  *“Yes, it is ignorance, they say the child is too young, and therefore, they don’t need to brush” FGD5 _Younger_FemaleP1*  *“…like an 18 months old chewing gum, it is bad. Then, children use a toothbrush that is not suitable for their ages, these are parts of the problems. FGD2_Older_ FemaleP7.* |
|  | Excessive consumption of sugary foods | *“Eating junks, always! especially the sugary ones… Whoever indulges in such habit will not likely escape dental problems and not just dental problems only, even the entire body…”. FGD4_Older_MaleP2* |
|  | Spiritual (supernatural) causes | *“Spiritual forces may be responsible for dental problems. When the deities are offended, they could attack one with cancer for example. May we not offend them” FGD4_Older_ MaleP7* |
|  | Hereditary or familial causes | *“As for me, I believe dental problems are inherited. If you look well, you will see a pattern in the family” FGD6_Younger_MaleP9* |
|  |  | *“I think dental problems run in the family” FGD5_Younger_FemaleP8* |
