# Supplementary material for: Oral health in an urban slum, Nigeria: residents’ perceptions, practices and care-seeking experiences
Source: BMC Oral Health. 2023 Sep 9;23:657. doi: 10.1186/s12903-023-03303-5 (PMC10492367; doi:10.1186/s12903-023-03303-5)
Supplement: Supplementary file 2 — Additional file 2. [file 12903_2023_3303_MOESM2_ESM.docx]

Appendix 2: The slum residents’ dental health care practices, relief remedies for dental ailments, reasons for the choice of remedy, and perceived consequences of self-care remedy options

| ***Theme*** | ***Sub-theme*** | ***Household representatives’ quotes*** |
| --- | --- | --- |
| ***Dental health care practices*** | **Hygiene practices** | *“A way we can care for our mouth is to brush the teeth twice daily” (FGD3_Younger_MaleP5).*  *“I believe for the children with bleeding gums, it is because they don’t clean their teeth well, that is why it is bleeding if they wash their teeth well, it will not bleed” FGD2_Older_FemaleP7* |
|  | **Teeth cleaning implements** | *“Some use “epa Ijebu” (a dentrifice), they use it to clean their teeth, while other people use ash, put it on their brush and clean their teeth with it” (FGD2_Older_FemaleP1)* |
| ***Relief remedies for dental ailments*** | **Self- medication** | *“For pain or swelling, we first use Beecham Ampiclox (an antibiotic), then if you can chew the white vitamin c on the part affected, it will dry up [the pain] (FGD1_Younger_FemaleP1).* |
|  |  | *“The paracetamol method is common too, our people, use novalgin, or paracetamol. Just place any of these on the aching tooth and that will be the end of discomfort” (FGD2_Older_FemaleP1)* |
|  |  | *“Codeine is also used” (FGD2_Older_FemaleP8)*  *Facilitator: the codeine you mentioned ma, how is it applied?*  *“Same way!* (referring to how the other medications were used), *just hold it on the aching teeth and that will be the end of it” (FGD2_Older_FemaleP8)* |
|  | **Other self-care practices** | *“I use hot water and salt solution, I put it on it (the tooth), then swallow paracetamol tablet, it gives relief” (FGD2_Older_FemaleP1)* |
|  |  | *“A child with bleeding gums for example, a way we can treat the bleeding is to brush the teeth twice daily and this will stop” (FGD5_Younger_FemaleP5)* |
|  |  | *“it is going to about two years now, when I have suffered toothache especially the one on this my front tooth. Although, I didn’t go to any hospital for medical treatment, someone recommend toothpaste (oral B) for me. I bought and applied it and it worked for me. Since then till now, I didn’t feel the pains again. FGD1_Younger_FemaleP8* |
|  |  | *"There are some children who have gum bleed problems, I usually tell them to use warm water and salt to gargle the mouth” FGD2_Older_Female_P1* |
|  |  | *“When you feel a pain on your tooth, you can apply the close up toothpaste and the pain will disappear, then maxima (toothpaste) is also good, it does same thing”. FGD2_Older_Female_P3* |
|  |  | *“Then there is cow's urine, you can also use it, you just put it on the teeth, it works for teeth problems” FGD6_Younger_Male_P2* |
|  |  | *“When I had a toothache, I used a mixture of regal gin, and “ata Ijosi” (dry pepper from Jos, Nigeria) and placed it just at the center of my aching tooth, held it there for a few minutes and the swelling burst and the pain disappeared. FGD3_Younger_Male P1* |
|  |  | *“A mixture of ‘Oral B’ (toothpaste), with a powdered tooth paste, like ‘Jim’ too helps to relieve dental pain” FGD5_Younger_Female_P10* |
|  |  | *“That herbal paste, it has been mentioned before, the powdered one in small containers. Errmm! It is called teething mixture; it works very well for any form of dental problem” FGD5_Younger_Female_P11* |
|  |  | *“Another one is ‘Ogun efu’. FGD2_Older_Female_P5*  *Facilitator: what is that, what does it look like and what is it used for?*  *“It is sold around by vendors. It is hot and spicy, it has items like alligator pepper, dry pepper, and some other things like that, and it also has gin, it is used for any mouth related disease, just hold the mixture in your mouth for a while, that’s all” FGD2_Older_Female_P8* |
|  |  | *“When I had dental pain, I used traditional soap (native black soap mixed with other things and usually prepared or prescribed by a traditional healer) to cure it. I didn’t go to any hospital. I only came here today because I was invited. It was then that I heard about the presence of a dental clinic around here for the first time. That is all”. FGD6_Younger_Male_P3*  *Facilitator: Sir, can you tell us what constitutes this traditional soap you used?*  *“Atare, ata ijosi, white alum, konafuru (cloves) and the usual black soap, ehen! (he affirms) you will mix them together to wash the teeth. FGD6_Younger_Male_P3*  He continued *“Another option is to mix ata ijosi dudu ti ko pon (unripe, dried pepper from Jos, Nigeria), with seaman (gin). Shake the mixture well and hold at the corner of your tooth. Do not swallow it oh (he warns), hold it there for some minutes then spit it out. FGD6_Younger_Male_P3* |
|  |  | *“When my brother had teeth problem a neighbour went all the way to IITA (an Institute) area to buy some plants. He mixed the plants with some other stuff and squeezed the combination to produce a liquid which he gave to my brother, he asked my brother to not swallow it o! (he stressed) that he should just hold it at the corner of the mouth where, it hurts. Then after thirty minutes when my brother spat out the liquid. I personally saw live worms crawling from what he spat out”. FGD6_Younger_Male_P4* |
|  | **Chemist / patent medicine store** | *“One can also use Ampiclox- the Beecham type o! (she stressed) for tooth problems, you can tell those chemist people, they know how to mix the medicines together”. FGD1_Younger_Female_P6*  Moderator: why Beecham Ampiclox?  *“It works better and faster” FGD1_Younger_Female_P6* |
|  | **Dental clinic facility** | *“If anyone had a tooth ache, we usually direct them to go to [names the location], immediately” FGD1_Younger_Female_P7*  *Facilitator: Where is [name of location]?*  *“the dental clinic place in this [name of location] community is the place called [names location], when people have dental issues, we direct them to the place. Even me that is the place I use” FGD1_Younger_Female_P9* |
|  |  | *… here, you know we are close to you, so it is different, we naturally go to [name of location] for our dental problems… FGD2_Older_FemaleP11* |
| ***Reason for choice of remedies*** | **immediate relief** | *“…when a pain becomes unbearable, the next thing is to find something, anything that can provide relief, our people believe it is in order to try out one of the things mentioned, first, in search of help, but if they don’t get the help they desperately need, then, they go on to other options, it is just for immediate relief” FGD2_Older_Female_P* |
|  | **affordability** | *“Since there is no money, there is nothing one can do, so we resort to taking alternatives like the ones mentioned, whose prices are a lot cheaper. So generally, it is money” FGD2_Older_Female_P1*  *“May we not experience any health challenge o (people chorused-Amen). But generally speaking, if a child is ill, everyone says ‘take care, go to the hospital’ but the mother of the child knows that she can’t afford the money to take the child to the hospital, someone may even offer to lend her some money, and some hospitals will not even accept the child until the deposit is fully paid. So this is why such a person will likely end up with the herb sellers and traditional healers since their charges are usually much less”. FGD5_Younger_Female_P1* |
| ***Consequences of use of self-care remedies for dental ailments.*** | **Not effective / of minimal effectiveness** | *You see those native herbs? It is deceitful when it comes to akokoro issues or anything that pertains to teeth problem. It just doesn’t work. It only provided me with transient relief. FGD4_Older_MaleP4* |
|  |  | *“I once used a substance from inside a bottle. I bought it from a “Hausa” (a major tribe in Nigeria) vendor. Hausas are the ones that usually sell it, I can’t remember its name now, but I know that “asa taaba” (a tobacco product) is one of the materials used to prepare it. You also hold it (the product) in your mouth, although they say it is very powerful but it didn’t work for me”. FGD6_Younger_MaleP7* |
|  |  | *…It works for some people but never worked for me. I think it is my body system. That is why I go to hospital to seek medical treatment for my teeth problems. FGD2_Older_FemaleP4* |
|  |  | *…I have used several native treatments which didn’t work, I ended up in a dental clinic that time after everything” FGD2_Older_FemaleP4* |
|  | **Potential to worsen health condition** | *“… when they come to us for some acid (works as battery charger) fluid to use for dental treatment, I personally always advise them not to use acid water because it can endanger their life. This acid can eat up their gums and cause serious damages to internal organs when accidentally ingested. I usually advise against its use”* ***FGD6_Younger_MaleP5*** |
|  | **Death** | *“I saw it (use of battery fluid) kill someone that had eye problem. He was advised to apply battery water to it. He woke up in heaven (died). Our people believe that battery water kills dental disease. But it is at a high risk. So, it not advisable to use battery water.*  ***FGD4_Older_MaleP1*** |
|  |  | *“… Yes, we are aware of it (use of battery fluid) but we don’t advise people to use battery water for any form of treatment because it has killed in the past. It is not the best solution****. FGD2_Older_FemaleP3*** |
