# Supplementary material for: Oral health in an urban slum, Nigeria: residents’ perceptions, practices and care-seeking experiences
Source: BMC Oral Health. 2023 Sep 9;23:657. doi: 10.1186/s12903-023-03303-5 (PMC10492367; doi:10.1186/s12903-023-03303-5)
Supplement: Supplementary file 3 — Additional file 3. [file 12903_2023_3303_MOESM3_ESM.docx]

Appendix 3: The slum residents’ treatment-seeking experience: the enablers and barriers to accessing care and suggested measures to improve timely access to care

| ***Theme*** | ***Sub-theme*** | ***Household representatives’ quotes*** |
| --- | --- | --- |
| **Enablers to seeking care** | **Warm reception** | *“My own experience was okay o! I didn’t have any further problem after the treatment… I brought one of my children to complain about his teeth. The doctor took us inside, removed the tooth and gave us some medicines to use. He also told us about using warm salt water solution. Since the treatment, there has never been any dental problem again with my child” FGD5_Younger_FemaleP9.* |
|  | **Prompt service** | *My own experience is that, when I got here I made my complaint about my teeth and they responded to it without delaying me and told me what I should do”. FGD4_Older_MaleP5.* |
|  |  |  |
| **Barriers to seeking care** | **Lack of money** | *“money is the main issue; it hinders us from seeking care from the dental care facilities”* ***FGD4_Older_MaleP7*** |
|  |  | *“…I brought someone here some time ago, he had an accident, the teeth were out of place, when I got him to dental clinic, the bill they gave us (quoted) was around #14,000 to #15,000. We tried to raise money as the boy hadn’t any money on him, the boy simply said, if he had any money on him, he would rather use it to feed. He resigned to his fate. Till now the young man is still going around with the teeth out of place like that… So there is no money!”* ***FGD4_Older_MaleP5*** |
|  | **Fear of perceived high treatment bills** | *“Or if the treatment can be free, it will make people to go the dental clinic more, because there will be no fear associated with expected treatment bill or thoughts of how much they will be asked to pay”* ***FGD2_Older_FemaleP2*** |
|  |  | *“Yes o! their bills around here (referring to the dental clinic) are too high for some of us to bear. The thoughts of it alone is discouraging. So they should please help us and do something about it.* ***FGD3_Younger_MaleP8****.* |
|  |  | *“the cost for tooth replacement is high and that is why I have not done it, I should have done it long ago because this one (tooth) is just there, nothing is supporting it again, money is the challenge, when we got there (dental clinic), the amount was beyond my reach, if you start it (prosthetic tooth services) up here and can do something about the cost, we will very much appreciate it.* ***FGD2_Older_ FemaleP11*** |
|  |  | *“Yes, o! you pay for many different things there. You pay for card, you pay to see doctor, you pay for treatment and you even pay for medicine. By the time you are out. Your pockets are empty”* ***FGD1_Younger_ FemaleP7.*** |
|  | **Fear of the pain from tooth extraction** | *“One of the challenges is the pain that one goes through during the tooth removal. The sight of the instruments alone is very scary. You get panic attacks frequently in that place (referring to the dental clinic)”* ***FGD4_Older_MaleP6*** |
|  | **Dissatisfaction with ‘extraction only’ service** | *“What we want the government to do for us is to ensure a way of treatment that will ease the teeth pain or that will cure it because we don’t want to remove our teeth again… the pain is too much. I have removed one of my teeth in the clinic here which I don’t want to remove it then. It is now affecting me even till now because another one didn't grow so if there is an alternative I think we will prefer it”.* ***FGD5_Younger_FemaleP9.*** |
|  | **Lack of awareness about the existence of a dental care facility in the community.** | *“Some people don’t know there is a place (referring to the dental clinic) like that here (referring to the community) except for those of us who live close by. For example, recently my sister came to me and said she was in pain from toothache and said she didn’t know where to go. I had to personally take her to the dental clinic for them to remove the tooth. So, some people are not aware of dental clinic presence in this community.* ***FGD5_Younger_FemaleP6*** |
|  |  | *“En.. before, we were not aware of its (referring to dental clinic) existence here. But now we know, we will patronize it****” FGD6_Younger_MaleP2*** |
|  | **Misinformation about the services offered in existing government clinic** | *Me I know there is a dental clinic, but I always thought the place was built for the benefit of our fathers and mothers. Whenever I or my family needed dental care, I usually go to either … or …. (named teaching hospitals or state hospital). To me, it didn’t appear they could offer much (referring to varied services). So I just never bothered visiting the place****. FGD 5_Younger_MaleP9*** |
|  |  | *“Is it not only teeth extraction they do there”* (participant teases) ***FGD5_Younger_FemaleP3*** |
|  |  | *“I believe this place (pointing at dental clinic) was built for the benefit of our fathers. As for me if I want to receive any treatment I will go to (the teaching hospital) simply because I am not aware that some treatments are available in the place”.* ***FGD3_Younger_MaleP2*** |
|  |  |  |
| **Suggested measures and recommendations to improve timely access to dental health care**. | **Wider range of treatment options** | *"We need dentists in this community. We know they are in ‘orita mefa’ (teaching hospital) and that place is too far for many of us to access. So we want more of them with different capabilities (sub-specialties) in our community”* ***FGD5_Younger_FemaleP4*** |
|  |  | *"As the matter of teeth replacement, what I mean is plastic teeth, they should be able to do that there (referring to the dental clinic- olokun) also, so that we don't think of going far for it"* ***FGD2_Older_FemaleP1*** |
|  | **Reduced cost or free care** | *“if the amount they charge can be reduced, you will see more people use the dental clinic, it is the money that has made people stay back… me in particular….if with just a little amount of money, you will get the care you need, people will come, and may we not be sick”* ***FGD1_Younger_ FemaleP9.*** |
|  |  | *“They should not bill so high, there is no way the money will not be there”.* ***FGD1_Younger_ FemaleP6*** |
|  | **Readily available medicines and drugs at discounted rates** | *“I want the government to provide enough drugs that we can use to treat our teeth. Make it available in all the hospitals especially in this our local clinic here in our community”.* ***FGD5_Younger_FemaleP4*** |
|  |  | *“We need drugs at discounted rates and lecture on this (dental health care) at least once in every three or six months”.* ***FGD2_Older_FemaleP11*** |
|  | **Need for more visibility** | *“Some people don’t know there is a place (dental facility) like this here, except for those of us who live close by….”* ***FGD1_Younger_FemaleP2****.* |
|  | **Oral health education talks** | *“We need awareness and educative lectures on oral health issues”.* ***FGD5_Older_FemaleP9*** |
|  |  | *“Some of us have not received any lecture on this before and we are interested”.* ***FGD6_Younger_MaleP9*** |
|  |  | *“the only thing is that I was not aware of what you said that we should come for our checkup at least once, in six months, we were not informed of this before”.*  ***FGD3_Younger_ MaleP8*** |
|  | **Community outreach programmes** | *“There was a time that the people (dental personnel) used to do community outreach, they go around from house to house to raise awareness, but it was over a long time ago”* ***FGD2_Older_FemaleP1*** |
|  |  | *“that is what I want to say too, that you should go out into the community, just the way you have come here,… to talk about oral health, and we honoured your invitation, if they can do that, more people will attend dental services, because some people may not know that this kind of thing is available”* ***FGD2_Older_FemaleP3*** |
|  |  | *“…So, my suggestion is that they should create awareness from house to house or street by street, informing people about this place and the kind of treatments they offer… “.* ***FGD3_Younger_MaleP2*** |
|  | **Periodic mouth**  **screening exercises at the community level** | *“What I want to say is that you know our teeth are very important to us so if the federal government can organize a program that will go around to do teeth checkup for us once in three months”.*  ***FGD4_Older_MaleP3*** |
|  |  | Interviewer: Do you want them to come to your house for the check-up or you won’t mind going to visit the nearest clinic for the check-up or what?  *“We will come to the hospital once we are notified or the awareness level is raised….”* ***FGD4_Older_MaleP3*** |
|  |  | ***“****We are supposed to have had this kind of meeting long before now”* ***FGD4_Older_MaleP7*** |
